# Seasonality and Geography Have a Greater Influence than the Use of Chlorine-Based Cleaning Agents on the Microbiota of Bulk Tank Raw Milk

**DOI:** 10.1128/AEM.01081-21

**Published:** 2021-10-28

**Authors:** Min Yap, David Gleeson, Paul W. O’Toole, Orla O’Sullivan, Paul D. Cotter

**Affiliations:** a Food Bioscience Department, Teagasc Food Research Centre, Fermoy, Ireland; b School of Microbiology, University College Cork, Cork, Ireland; c Teagasc Animal and Grassland Research and Innovation Centre, Fermoy, Ireland; d APC Microbiome Ireland, Cork, Ireland; University of Helsinki

**Keywords:** DNA sequencing, dairy, metagenomics, microbiota

## Abstract

Cleaning of the production environment is vital to ensure the safety and quality of dairy products. Although cleaning with chlorine-based agents is widely adopted, it has been associated with detrimental effects on milk quality and safety, which has garnered increasing interest in chlorine-free cleaning. However, the influence of these methods on the milk microbiota is not well documented. This study investigated the factors that influence the raw milk microbiota, with a focus on the differences when chlorine-based and chlorine-free cleaning of milking equipment are used. Bulk tank raw milk was sampled during three sampling months (April, August, and November), from farms across Ireland selected to capture the use of different cleaning methods, i.e., exclusively chlorine-based (*n* = 51) and chlorine-free cleaning (*n* = 92) and farms that used chlorine-free agents for the bulk tank and chlorine-based cleaning agents for the rest of the equipment (*n* = 28). Shotgun metagenomic analysis revealed the significant influence of seasonal and geographic factors on the bulk tank milk microbiota, indicated by differences in diversity, taxonomic composition, and functional characteristics. Taxonomic and functional profiles of samples collected in November clustered separately from those of samples collected in other months. In contrast, cleaning methods only accounted for 1% of the variation in the bulk tank milk bacterial community, and samples collected from farms using chlorine-based versus chlorine-free cleaning did not differ significantly, suggesting that the chlorine-free approaches used did not negatively impact microbiological quality. This study shows the value of shotgun metagenomics in advancing our knowledge of the raw milk microbiota.

**IMPORTANCE** The microbiota of raw milk is affected by many factors that can control or promote the introduction of undesirable microorganisms. Chlorine-based cleaning agents have been commonly used due to their effectiveness in controlling undesirable microorganisms, but they have been associated with the formation of chlorine residues that are detrimental to product quality and may impact consumer health. Chlorine-free alternatives have been recommended in some countries, but the influence of cleaning agents on the milk microbiota is unknown. Here, we investigated the influence of cleaning methods and other factors on bulk tank raw milk. Results showed that season and location had a greater influence on the milk microbiota than the cleaning agents used. Indeed, the similar microbiota compositions of raw milk from farms that used chlorine-based and those that used chlorine-free cleaning methods supports the further use of chlorine-free cleaning agents in dairy production.

## INTRODUCTION

The raw milk microbiota is complex, with many factors contributing to its composition (1). Understanding the factors that positively or negatively impact the microbiota of raw milk is important, as it can affect the safety and quality of foods produced. Cleaning of equipment is one important aspect of food processing that, if done inefficiently, may cause the unwanted transfer of microorganisms from food production and processing surfaces to product. Chlorine-based cleaning products have been commonly used in many industries, such as agriculture, water treatment, and food processing, due to their efficient killing of a broad range of microorganisms (2), including by disrupting protein synthesis and by increasing cell membrane permeability (3, 4). In dairy production and processing, chlorine, in the form of hypochlorites or gaseous chlorine, is widely used in cleaning products for the disinfection of equipment (2, 5, 6). However, the use of chlorine has been associated with the formation of total organic chlorine residues, which have been found to affect the end product quality and may pose a health risk to consumers (7). Such chlorine residues include trichloromethane, which has been classified as a potential carcinogen, and chlorite, chlorate, and perchlorate, which have been found to cause oxidative stress in cells and/or to cause thyroid dysfunction (6–8). As a result, alternative cleaning methods involving the use of chlorine-free detergents and sanitizers have been recommended as substitutes. In this regard, it is notable that chlorine-free detergents and a detergent containing sodium hypochlorite resulted in similar posttreatment levels of bacteria, as determined by culture-based approaches, in bulk tank milk (9). However, other culture-based investigations have revealed that chlorine-based cleaning more effectively reduced levels of spore-forming bacteria than chlorine-free (10) or mechanical cleaning (11). Greater resolution of the influence of the cleaning detergents used in dairy production on the microbiological community in raw milk will help determine if the removal of chlorine-based agents impacts milk quality.

High-throughput sequencing has been used to characterize the milk microbiome, including to identify mastitis-associated pathogens and to assess the safety and quality of milk for downstream use in dairy products (12). While amplicon sequencing, such as the targeting of the 16S rRNA gene, has been most widely used, the greater taxonomic resolution, to species level, afforded by shotgun metagenomic sequencing is needed to distinguish harmless and undesirable taxa from the same genus (13) and to facilitate further characterization of the functional profiles of the microbiota, including antibiotic resistance and toxin determinants (14). Regardless of the approach taken, studies have revealed that the composition of bulk tank milk is significantly affected by many factors, including season, lactation stage, farm system, feed, and others (15, 16). The fluctuating abundances of several taxa based on season can have important implications for the quality and safety of milk (17–19). Moreover, different farm management practices and other environmental factors also influence the microbiota of raw milk (20). Understanding these factors and their influence on the raw milk microbiota can help inform food safety and quality decisions for downstream processing. This present study was run concurrently with that of D. Gleeson, L. Paludetti, B. O’Brien, and T. Beresford (submitted for publication), which investigated samples under the same experimental conditions using culture-based and chemical analyses. The aim of this study was to determine the factors influencing the bulk tank raw milk microbiota using shotgun metagenomics, with particular focus on differences in taxonomic or functional characteristics associated with the use of chlorine-based and chlorine-free cleaning agents.

## RESULTS

### Sampling month and location had greater influence on the taxonomic diversity and profiles of the bulk tank milk microbiota than cleaning method.

A total of 171 raw milk samples collected from 57 bulk milk tanks across Ireland were sampled over each of the three sampling months. Some farms changed their cleaning routine over the sampling months and, as a result, 51 samples were from farms that used chlorine-based agents in their cleaning routine (C), 92 samples were from farms that used a chlorine-free cleaning routine (CF), and 28 samples were from farms that used chlorine-free agents for bulk tank cleaning and chlorine-based cleaning methods for the rest of the equipment (BTCF). There were varied sample sizes between sampling locations (A, *n* = 45; B, *n* = 45; C, *n* = 57; D, *n* = 24). Further details on the breakdown of samples are given in Table S1 in the supplemental material. Shotgun metagenomic sequencing of the raw milk samples generated an average of 7,204,026 (±1,759,482) high-quality paired-end reads per sample. A high proportion (mean, 98.01%; range, 82.1 to 99.5%) of metagenomic reads sequenced were host reads that aligned to the Bos taurus genome. An average of 62,976 (±106,273) reads were assigned as nonhost reads (mean, 1.99%; range, 0.49 to 17.9%).

**FIG 1:**
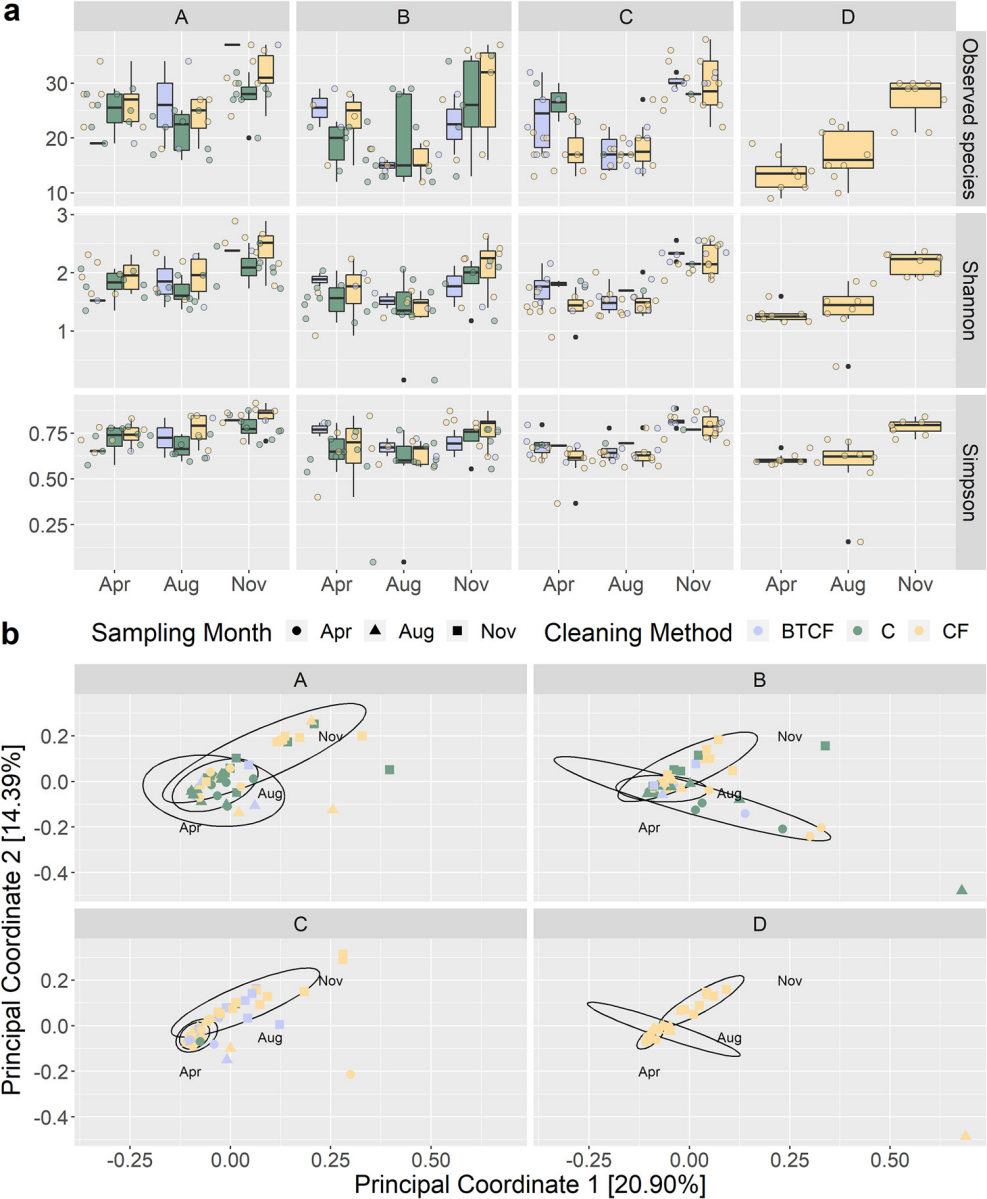
Diversity analysis of the microbiota of bulk tank milk samples. (a) Observed species, Shannon, and Simpson alpha diversity analysis of bulk tank milk samples. (b) Bray-Curtis principal-coordinate analysis (PCoA) plots, with ellipses representing clustering by sampling month. Samples are from farms taken in 3 different sampling months—April, August, and November—from 4 different sampling locations (designated A to D). Samples are classified in 3 different groups of cleaning methods, with samples from farms where chlorine agents are used for cleaning bulk tanks only (BTCF) and from farms which exclusively use chlorine (C) or exclusively use chlorine-free agents (CF) in their cleaning routines.

The alpha diversity (observed species and Shannon and Simpson metrics) of the bulk tank milk microbiota was not significantly different across samples from the 3 different cleaning methods (Fig. 1a). Compared by sampling month, the alpha diversity values of raw milk sampled in April and August were not significantly different, and both were significantly lower (*P < *0.001), across all three metrics used, than the alpha diversity of raw milk sampled in November. Between locations, the alpha diversity of location A was significantly higher than those of locations B and D by all 3 metrics and that of location C by Simpson score (*P < *0.05). For beta diversity, samples did not cluster based on cleaning method, which was found to only account for 1.6% of the variation of the bacterial community (Fig. 1b and Table 1). On the other hand, significant differences were apparent by sampling month, where November samples clustered distinctly apart from the April and August samples. Similarly, based on sampling location, location B samples clustered separately from those from other locations. Both sampling month and location contributed significantly to the variation in microbiota composition (*P < *0.01), accounting for 10.1% and 6.7%, respectively (Table 1).

**TABLE 1:** PERMANOVA of taxonomic composition and functional properties (based on results from SUPER-FOCUS) of the bulk tank milk microbiota

Model or variable	*R* ^2^	*P*
Taxonomic composition		
Cleaning method	0.016	0.069
Sampling month	0.106	0.001
Sampling location	0.067	0.001
Cleaning method*sampling month	0.014	0.901
Cleaning method*sampling location	0.020	0.389
Sampling month*sampling location	0.054	0.001
Cleaning method*sampling month*sampling location	0.036	0.55
Residuals	0.687	
Functional profiles		
Cleaning method	0.011	0.477
Sampling month	0.041	0.001
Sampling location	0.037	0.003
Cleaning method*sampling month	0.017	0.859
Cleaning method*sampling location	0.021	0.541
Sampling month*sampling location	0.043	0.064
Cleaning method*sampling month*sampling location	0.040	0.661
Residuals	0.790	

Given the possibility of the cleaning method confounding the impact of sampling month and location, results were stratified into the 3 cleaning methods, and diversity analysis and permutational analysis of variance (PERMANOVA) were done to determine the effect on community structure. November samples, irrespective of cleaning method, had significantly higher alpha diversities and clustered separately from April and August samples (*P < *0.05) (Fig. S1). In terms of sampling location, alpha diversity was higher for location A across all cleaning methods, and particularly significantly higher for all metrics in farms that used chlorine-free cleaning routines (Fig. S1). Location B samples clustered slightly further away from samples from other locations, with significant differences between locations found for the other 2 cleaning methods apart from samples from farms that used chlorine-based agents (Table S2).

**FIG 2:**
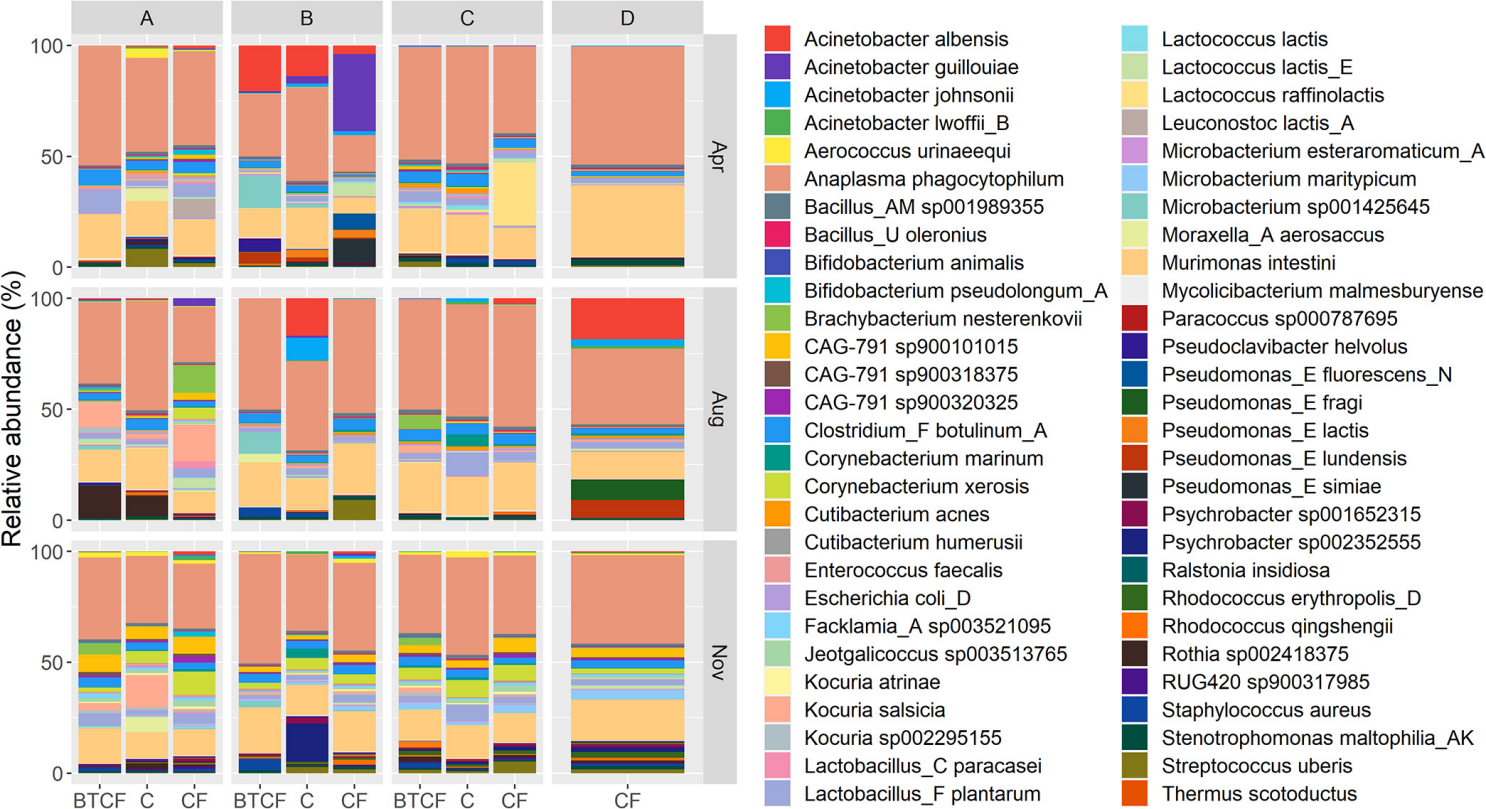
Taxonomic profiles of the bulk tank milk microbiota at relative abundances greater than 0.01%. Samples were taken during 3 different sampling months—April, August, and November—from 4 different sampling locations (designated A to D). Samples are classified in 3 different groups of cleaning methods, with samples from farms where chlorine agents are used for cleaning bulk tanks only (BTCF) and from farms which exclusively use chlorine (C) or exclusively use chlorine-free agents (CF) in their cleaning routines.

A total of 56 species were present at average relative abundances greater than 0.01% across all samples, as shown in Fig. 2. Anaplasma phagocytophilum, Acinetobacter albensis, and Murimonas intestini were the three most abundant species in all samples. No differences were found in the species abundances between cleaning methods, but significant differences between sampling months were found in 27 species and between sampling locations in 24 species (*P < *0.05) (Fig. S2 and Tables S3 and S4). Additional analysis for indicator species was performed to determine if any particular species was significantly associated with any cleaning method, sampling month, or sampling location. While no species were found to be associated specifically with any cleaning method, 16 species were associated with at least one sampling month, and 14 species were associated with at least one sampling location. For sampling months, Ralstonia insidiosa and Microbacterium esteraromaticum_A were associated with April (*P < *0.05), and Corynebacterium xerosis, CAG-791 sp900101015, Psychrobacter sp002352555, Microbacterium maritypicum, Aerococcus urinaeequi, Jeotgalicoccus sp003513765, CAG-791 sp900320325, Psychrobacter sp001652315, Rhodococcus erythropolis_D, Kocuria sp002295155, CAG-791 sp900318375, Facklamia_A sp003521095, RUG420 sp900317985, and Kocuria atrinae were associated with November samples (*P < *0.05). For sampling locations, CAG-791 sp900101015, Aerococcus urinaeequi, Escherichia coli_D, Enterococcus faecalis, CAG-791 sp900318375, Facklamia_A sp003521095, Paracoccus sp000787695, RUG420 sp900317985, and Lactobacillus_C paracasei were associated with location A (*P < *0.05) and Anaplasma phagocytophilum, Clostridium_F botulinum_A, Bacillus_AM sp001989355, Microbacterium esteraromaticum_A, and Mycolicibacterium malmesburyense with location C (*P < *0.05). No particular species were specifically associated with locations B and D, though Acinetobacter spp. were detected in greater relative abundances in both of these locations.

Climate data on the temperature, rainfall, and wind from sampling days was retrieved in an effort to correlate these climatic data with seasonal and geographical differences (Table S5). Analysis indicated that these climatic factors significantly influenced the microbiota (adjusted *R*^2^, 0.0785; *P < *0.01). Redundancy analysis showed that in terms of the climatic factors, the locations only differed slightly and still clustered closely together (Fig. S3). Locations B and D cluster more closely together and separately from A and C, although none of the climatic factors analyzed can visibly account for this.

### Similarly, functional characteristics of the bulk tank raw milk microbiome were influenced by sampling month and location.

**FIG 3:**
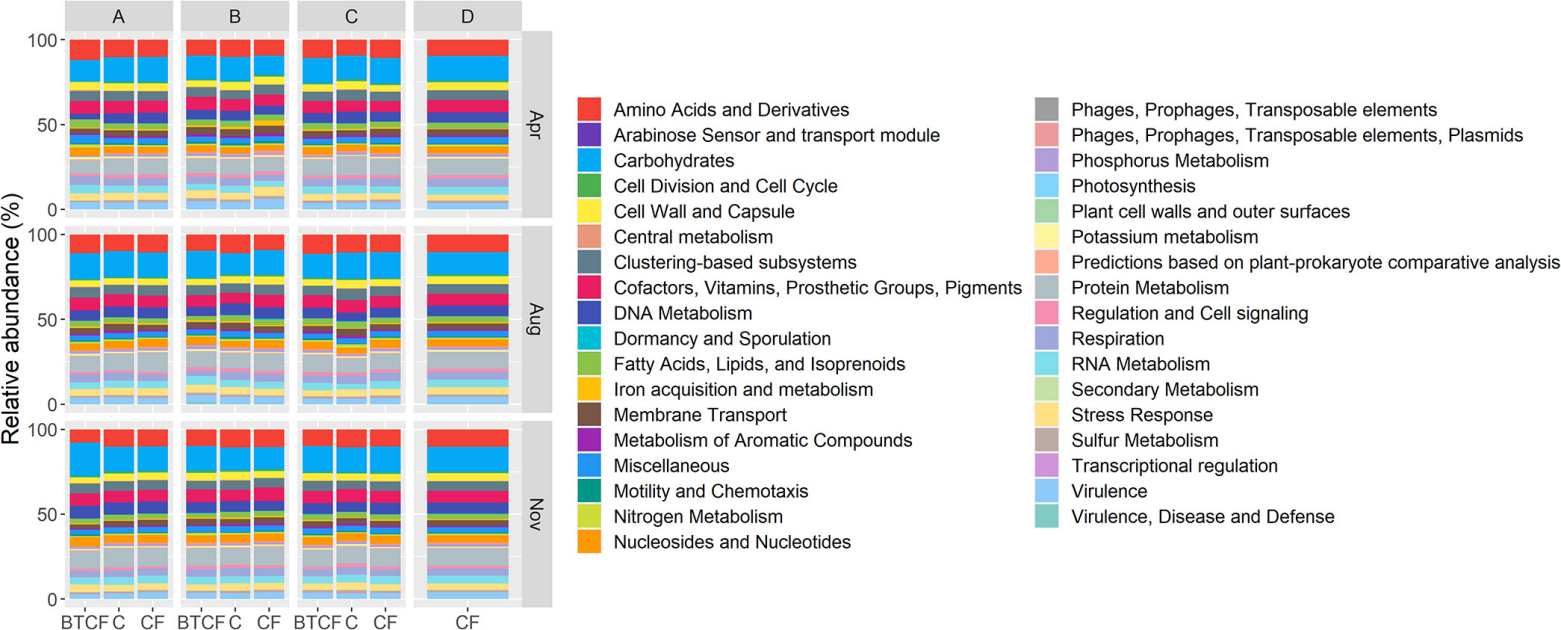
Functional profiles of the bulk tank milk microbiota, based on SUPER-FOCUS subsystem level 1 functions. Samples were taken during 3 different sampling months—April, August, and November—from 4 different sampling locations (designated A to D). Samples are classified in 3 different groups of cleaning methods, with samples from farms where chlorine agents are used for cleaning bulk tanks only (BTCF) and from farms which exclusively use chlorine (C) or exclusively use chlorine-free agents (CF) in their cleaning routines.

The functional potential of bulk tank milk microbiomes was predicted using SUPER-FOCUS, with genes involved in the metabolism of carbohydrates, proteins, and amino acids and derivatives being most abundant (Fig. 3). Genes associated with oxidative stress response, such as glutathione-related genes or those with protection from reactive oxygen species, are potentially related to chlorine resistance. These genes were detected at very low relative abundances (0 to 0.1%), with no significant differences detected between any cleaning method, sampling month, or sampling location. No genes encoding proteins that respond to reactive chlorine species were found in any samples. Overall, no significant differences in functional potential on the basis of cleaning method were found. Between sampling months, a total of 11 functional groups at subsystem level 1 significantly differed (*P < *0.05) (Fig. S4 and Table S6). Significant differences were found between sampling months for 28 functional groups at subsystem level 2 and for 98 groups at level 3 (*P < *0.01) (Table S6). Abundances of genes related to protein metabolism were seen to gradually increase, while those related to carbohydrates and fatty acids, lipids, and isoprenoids gradually decreased over time (Table S6). Between sampling locations, no differences were found at subsystem levels 1 and 2. However, at level 3, significant differences in abundances were noted for 1 functional group, the ESAT-6 protein secretion system in mycobacteria (locus ESX-1), which was significantly higher in location A samples (0.0367%) than in those from the other locations (B, 0.0148%; C, 0.0129%; D, 0.0075%).

### Higher numbers of antibiotic resistance gene marker reads were mapped to samples from farms which used chlorine-based cleaning agents versus those that used other cleaning methods.

**FIG 4:**
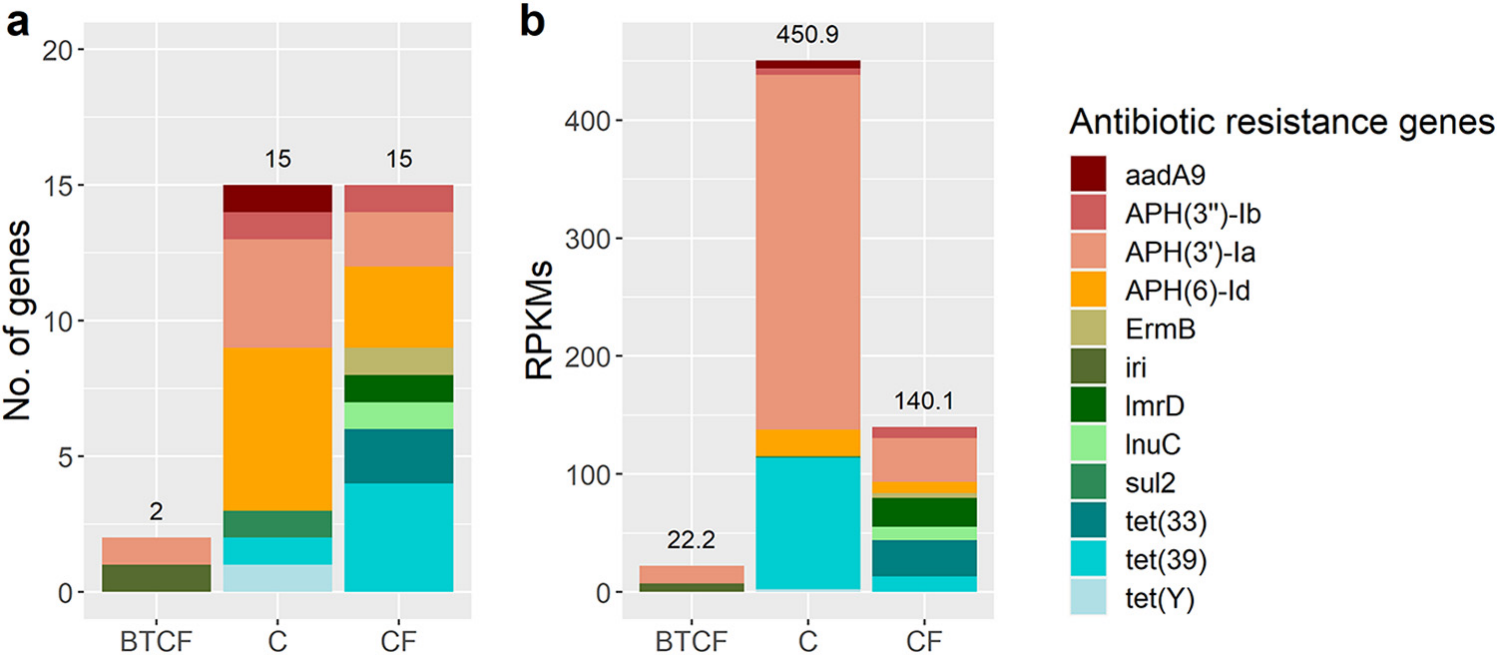
Antibiotic resistance genes (ARGs) found in milk samples from farms with different cleaning methods, expressed as number of genes detected (a) and normalized reads per kilobase per million reads (RPKMs) (b). Samples are classified in 3 different groups of cleaning methods, with samples from farms where chlorine agents are used for cleaning bulk tanks only (BTCF) and from farms which exclusively use chlorine (C) or exclusively use chlorine-free agents (CF) in their cleaning routines.

Genes encoding antibiotic resistance were identified using ShortBRED, and within bulk tank milk samples, 32 unique antibiotic resistance gene (ARG) markers were detected. These gene markers were found in 19 milk samples (11.1% of all milk samples). Antibiotic resistance gene markers corresponded to 6 different antibiotic classes, i.e., tetracycline, lincosamide, aminoglycoside, sulfonamide, rifampin, and erythromycin. Although the same numbers of genes were detected between samples from farms that used chlorine-free cleaning and chlorine-based cleaning (Fig. 4a), higher reads per kilobase of reference sequence per million sample reads (RPKM) were seen in milk from farms that used chlorine-based cleaning agents (Fig. 4b). Although not statistically different, average reads mapped to samples from farms that used chlorine-free agents for bulk tank cleaning alone were lower (11.12 ± 5.27 RPKM) than those for samples from farms that used a chlorine-free cleaning routine (9.34 ± 8.90 RPKM) and samples from farms that used chlorine-based agents in the cleaning (30.06 ± 49.30 RPKM).

## DISCUSSION

Here, we investigated the factors influencing the bulk tank milk microbiota, and particularly whether there were taxonomic and functional differences in the bulk tank raw milk microbiota associated with the use of chlorine-free or chlorine-based cleaning agents. Milk samples were collected across three sampling months and four different sampling locations and their microbiome profile assessed. Overall, taxonomic profiles of the raw milk sampled were somewhat different from those previously reported in other studies on the raw milk microbiome. Acinetobacter, Lactococcus, Streptococcus, Corynebacterium, and Pseudomonas have frequently been reported as the most commonly detected genera in raw milk that are present in high relative abundances (12). Although these genera were among the top 10 detected in this study, Anaplasma and Murimonas were also found in high relative abundances. Both *Anaplasma* and *Murimonas* are host related, and therefore their presence is not entirely unexpected (21, 22). The identification of *Anaplasma* in raw milk had been previously reported, as several species have been known to cause disease in cattle (23). *Murimonas* is part of the family Lachnospiraceae, which has been commonly found in bovine milk and dairy farm environments (22, 24), although few studies have assigned a particular genus or species. The improved taxonomic assignment of shotgun sequencing could be the reason for the identification of Murimonas intestini in bulk tank raw milk samples in this study.

The characterization of the microbiota revealed similar taxonomic compositions and functional profiles regardless of the cleaning method used, which only accounted for 1% of the variation in the bulk tank milk bacterial community. The results provide added evidence that support previous culture-based investigations by Gleeson et al. (9), showing that the use of chlorine or nonchlorine cleaning does not affect the composition of raw milk. Interestingly, the taxonomic profiles and diversities of sampling locations B and D were more similar to each other than to those of other locations, despite B having a mix of samples from farms that used all three cleaning methods and D comprising only farms that used chlorine-free cleaning. This result further shows that cleaning method (the use of chlorine or chlorine-free cleaning agents) does not significantly impact the milk microbiome, and other factors have a greater influence, as evidenced by cleaning method accounting for a small percentage of the variation in taxonomic and functional profiles that was not found to be statistically significant (Table 1).

Seasonality is known to impact the microbiota composition of raw milk, where distinct seasonal differences in diversity and composition have been noted (17, 18, 25). In the current study, seasonality had a significant influence on the raw milk microbiota, accounting for 10% of the variation in microbiota composition, with significant effects of time also found in culture-based analysis of the same set of samples done in the concurrent study by Gleeson and colleagues (9). In Ireland, as milk production is based on seasonal calving, the collection dates corresponded to spring/early lactation (April), summer/midlactation (August), and winter/late lactation (November). Samples taken in November had the greatest diversity and a distinct microbiota composition compared to those of samples taken in April and August, corresponding to results previously reported (16). Although other studies completed in the United States and China noted a higher alpha diversity in spring/summer, this could be due to different farming practices, most notably Ireland’s predominantly pasture-based system, and the diverse nature of the raw milk microbiota (15, 18). Despite this, some similarities in specific taxa were evident, such as the increased abundance of species from the family Lachnospiraceae and phylum Actinobacteria in winter (15, 16). Additionally, the species identified from indicator species analysis that were associated with November samples were mainly host-related species, which reflect Irish farming practices in which cows are kept indoors in winter, which has been reported to impact the raw milk microbiota (20). Functional genes related to the nutrient composition of milk were found to gradually increase (protein metabolism) or decrease (carbohydrates and fatty acids), which indicated the influence of seasonality or lactation stage on the milk microbiota and subsequently on nutrient composition.

Together with differences across sampling months, differences in the raw milk microbiota were found across sampling locations, which was expected because geographical location and associated environmental and farming practices have previously been shown to influence milk microbiota profiles (26, 27). In this study, analysis revealed that sampling location had a significant influence on the bulk tank raw milk microbiota (Table 1). As evidenced in the results, the microbiota of raw milk from locations B and D were more similar to each other than to those from the other locations, although no definite correlation with climatic data was found (Fig. 2; see also Fig. S3 in the supplemental material). More information is required to better ascertain the influence of location on the milk microbiota. Farm practices, environment conditions, animal health, and diet are some factors that previous studies have found to be contributing factors (18, 20).

Besides taxonomic profiling that adds support to previous culture-based studies, sequencing-based methods allows for further characterization of the raw milk microbiota. Access to shotgun metagenomic sequencing facilitated functional profiling, with significant differences again being evident between sampling months and locations and not between cleaning methods. In terms of antibiotic resistance, results in this study show that milk is a reservoir of antibiotic resistance genes, which may proliferate in milk and be transferred to surfaces during transport or downstream processing. The most abundant ARGs detected in the milk samples were those that confer resistance to aminoglycosides and tetracycline and which have been previously reported in raw milk (28, 29). Notably, Acharya et al. (30) found that even after effective disinfection, bacterial cells and their DNA can persist in samples, which could result in potential reservoirs for ARGs. Furthermore, incubation at room temperature for short periods of time has been found to enrich ARGs in milk, which highlights the importance of proper cold-chain practices (29). It has also been reported that chlorination can enrich ARGs in drinking water (31, 32), and it has been found to promote the horizontal transfer of plasmids by natural transformation, which result in the exchange of ARGs across bacterial genera (33). These phenomena may explain why numerically more ARGs were detected in raw milk from farms that used chlorine in their cleaning routine. Additionally, while more research is needed for a deeper understanding of the resistome of bulk tank milk and the implications of the presence and contribution of ARGs to antimicrobial resistance along the dairy processing chain, this study demonstrates the use of sequencing as a potential tool for monitoring ARGs without the isolation and culture of bacteria, which is valuable in terms of food safety.

It should be noted that while this study provides a deeper understanding of the raw milk microbiota and its relationship with cleaning and other environmental or climatic factors, it has some limitations. First, sampling was performed on single days in each month, and so we cannot conclusively state that the patterns are representative of broader seasonal differences, although the patterns are consistent with previous studies. In addition, although shotgun metagenomic sequencing is a valuable tool in the characterization of the raw milk microbiota, it does not differentiate between viable and nonviable microorganisms. This issue, however, is of greater concern when dealing with samples with high levels of intact DNA from dead bacteria, such as recently pasteurized milk. Finally, the shotgun approach involves sequencing all DNA present in samples, which has resulted in the identification of high proportions of host DNA in each sample and low microbiological sequencing depth, which may have hindered the further resolution of taxonomic, functional, and antibiotic resistance profiling, as previously shown in other studies (34, 35). Since the conclusion of this study, we have investigated approaches to overcome this problem (36) that we will utilize in future studies.

Despite these challenges, the study clearly reveals that season and location have greater impacts on the bulk tank raw milk microbiota than the use of chlorine or chlorine-free cleaning methods. Our findings also show that chlorine-free cleaning methods are comparable with chlorine cleaning with respect to the net microbiota composition and its functional potential and thus provide further reassurance when considering chlorine-free approaches. Furthermore, this study shows the value that shotgun metagenomic sequencing adds in advancing our understanding of the raw milk microbiota.

## MATERIALS AND METHODS

### Sample collection and preparation.

Raw bovine milk samples (100 ml) were collected from bulk milk tanks from farms across Ireland on single days in April, August, and November 2019. Commercial farms, located mainly in the southern region of Ireland, were identified by 4 milk processors (locations A to D) to take part in the study. Farmers agreed to comply with the following three different cleaning methods: the use of chlorine-free cleaning products for the bulk tank only (BTCF), chlorine-free cleaning methods throughout the system (CF), and traditional chlorine cleaning protocols (C) throughout the study period. Chlorine-free cleaning involved the use of hot water washes with acid, with five suggested protocols available online for farmers to use for implementation (https://www.teagasc.ie/media/website/animals/dairy/research-farms/Chlorine-free-wash-routines_2020.pdf). The samples were transported in a cooler box to the laboratory and prepared as follows: 30 ml of the bovine milk sample was centrifuged at 4,500 × *g* for 20 min at 4°C. After centrifugation, the cream and supernatant were discarded, and the pellets were subjected to two washing steps, whereby the pellets were resuspended in sterile phosphate-buffered saline (PBS) and centrifuged at 13,000 × *g* for 1 min, after which the supernatant was discarded and the pellet used for DNA extraction.

### DNA extraction.

DNA from samples was extracted on the same day of sample collection. Pellets from the removal of cream were resuspended in 800 μl CD1 solution from the DNeasy PowerSoil Pro kit. From this point, extraction was carried out according to the manufacturer’s instructions (Qiagen, West Sussex, United Kingdom). The lysis step was performed using a TissueLyser II (Qiagen) for 10 min at 30 Hz, and DNA was eluted in 70 μl and stored at −20°C until use in library preparation.

### DNA library preparation and shotgun metagenomic sequencing.

DNA was quantified using the Qubit double-stranded DNA (dsDNA) high-sensitivity assay kit (Bio-Sciences, Dublin, Ireland). All samples were prepared for shotgun metagenomic sequencing according to Illumina Nextera XT library preparation kit guidelines, with the use of unique dual indexes for multiplexing with the Nextera XT index kit (Illumina). Following indexing and cleanup, samples were pooled to equimolar concentration of 1 nM. Samples were sequenced on an Illumina NextSeq 500 sequencing platform with a v2 kit, at the Teagasc DNA Sequencing Facility, using standard Illumina sequencing protocols. Sequencing controls and negative controls were used to ensure consistency between runs, and in total, 181 samples were sequenced (3 time points for each of 57 farms plus controls).

### Bioinformatic analysis of shotgun metagenomic data.

Raw metagenomic shotgun reads were quality checked and trimmed with Cutadapt (v1.18) and FastQC (v0.11.8). Reads were then aligned to the bovine genome (Bos taurus) to determine the number of host reads using Bowtie 2 (v2.3.4). Kraken2 was used for taxonomic classification of sample data using the Genome Taxonomy Database (release 89), which contains bacteria and archaea (37–39). SUPER-FOCUS (40) was used to characterize the microbiological functional potential of shotgun reads through the alignment of reads against a reduced SEED (41) database using DIAMOND (42). Antibiotic resistance genes were quantified using ShortBRED, which maps shotgun reads against markers from the Comprehensive Antibiotic Resistance Databases (CARD) (43), and results are expressed as normalized reads per kilobase per million reads (RPKM) (44).

### Climate data.

Data on the rainfall (mm), maximum and minimum temperatures (°C), grass minimum temperature (°C), and mean wind speed (knots) from weather stations that were representative of the four sampling locations on the three sampling days were retrieved from the Irish Meteorological Service website (www.met.ie).

### Statistical analysis and data visualization.

Statistical analysis and data visualization were performed in R (v3.6.3) (45). Kruskal-Wallis and pairwise Wilcoxon rank sum tests with the Benjamini-Hochberg *P* value correction was used for comparison between cleaning methods, sampling months, and sampling locations. Diversity analysis was done using the *vegan* package (46), with alpha diversity calculated as observed species, Shannon, and Simpson metrics and beta diversity as Bray Curtis metrics, visualized in a principal coordinate analysis plot. The “adonis” function from the *vegan* package was used to determine differences in composition of the community between groups of samples (number of permutations = 999). Redundancy analysis (RDA) was performed using the *vegan* package to determine differences in the effects of weather variables on microbiota composition between sampling locations. The “multiplatt” function from the *indicspecies* package was used to find species that were significantly associated with particular cleaning methods, sampling, months and sampling locations, by calculating Pearson’s phi coefficient of association and correcting for unequal group sizes using the parameter “r.g” (47). Data cleaning and analysis and visualization was done using *tidyverse*, *ggplot2*, *ggord*, and *ggpubr* packages (48–50).

### Data availability.

Sequence data generated during the current study have been deposited in the European Nucleotide Archive under accession number PRJEB42046.
