# Supplementary material for: Seasonality and Geography Have a Greater Influence than the Use of Chlorine-Based Cleaning Agents on the Microbiota of Bulk Tank Raw Milk
Source: Appl Environ Microbiol. 2021 Oct 28;87(22):e01081-21. doi: 10.1128/AEM.01081-21 (PMC8552886; doi:10.1128/AEM.01081-21)
Supplement: Supplemental file 1 — Tables S1 to S6, Fig. S1 to S4. Download AEM.01081-21-s0001.pdf, PDF file, 0.8 MB [file aem.01081-21-s0001.pdf]

SUPPLEMENTAL MATERIAL

TABLE S1. Sample numbers for each cleaning method according to sampling location (A to D), where samples are from farms that used chlorine-free agents for their bulk tank alone (BTCF), farms that used chlorine-based agents in their cleaning routine (C) and farms that used a chlorine-free cleaning routine (CF).

Cleaning Methods	Sampling Location				
	A	B	C	D	Total
C	24	23	4	-	51
CF	17	16	35	24	92
BTCF	4	6	18	-	28
Total	45	45	57	24	171

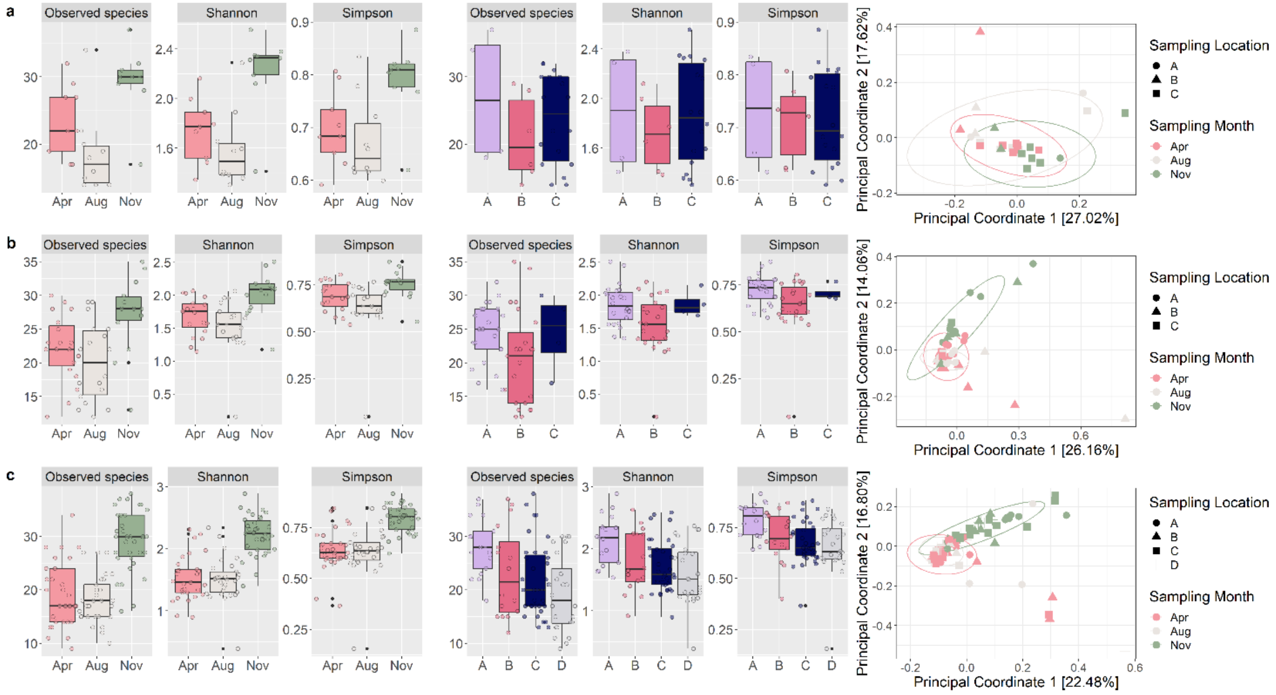

FIG S1. Alpha and beta diversity of raw milk stratified by cleaning method. Samples are from (a) farms that used chlorine-free agents for their bulk tank alone (BTCF), (b) farms that used chlorine-based agents in their cleaning routine (C) and (c) farms that used a chlorine-free cleaning routine (CF). Samples are from farms from 3 different sampling months – April (Apr), August (Aug) and November (Nov) from 4 different sampling locations (A to D).

TABLE S2. PERMANOVA analysis of taxonomic composition and functional properties (based on results from SUPER-FOCUS) of the bulk tank milk microbiota stratified by cleaning method. Samples are from farms that used chlorine-free agents for their bulk tank alone (BTCF), farms that used chlorine-based agents in their cleaning routine (C) and farms that used a chlorine-free cleaning routine (CF).

**Cleaning Method**		R^2	**P value**
BTCF	Sampling month	0.167	0.001
	Sampling location	0.150	0.005
	Residuals	0.684	
C	Sampling month	0.099	0.001
	Sampling location	0.047	0.201
	Residuals	0.853	
CF	Sampling month	0.124	0.001
	Sampling location	0.099	0.001
	Residuals	0.777	

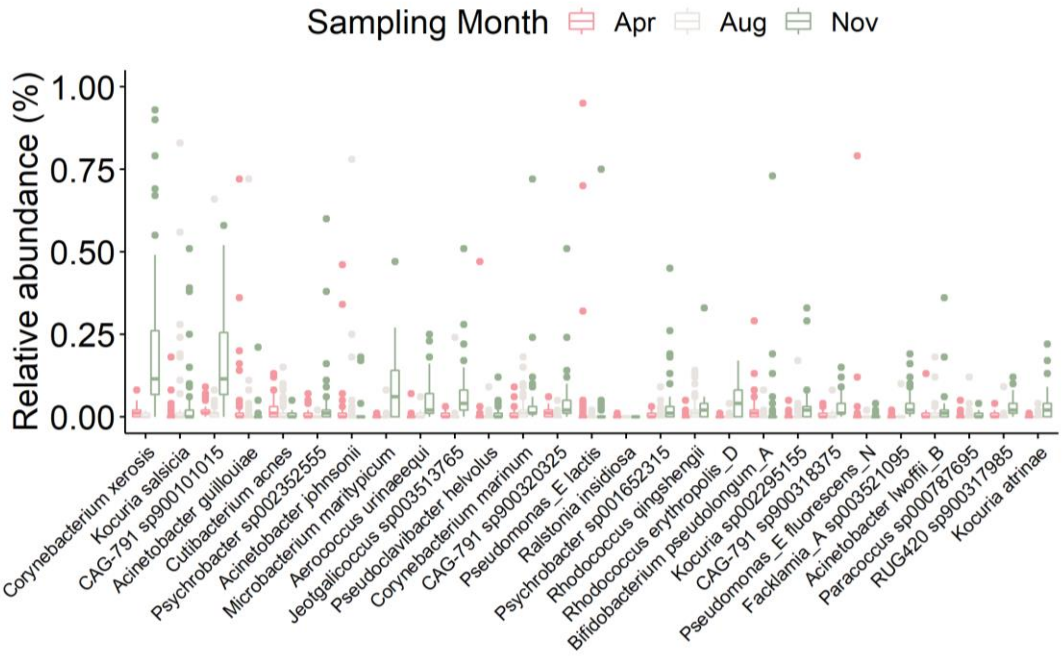

b

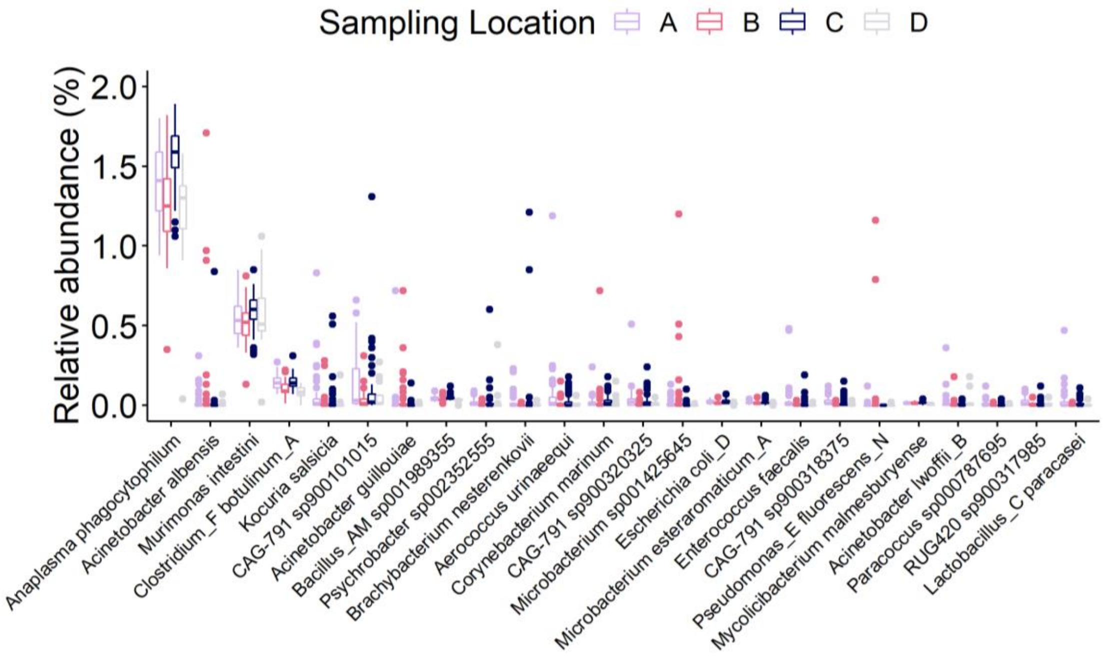

FIG S2. Significantly differential relative abundances of species that differed between (a) sampling month – April (Apr), August (Aug) and November (Nov), and (b) sampling location (locations A to D).

TABLE S3. Relative abundances of species that differed by sampling month – April (Apr), August (Aug) and November (Nov).

Species	Sampling Month		
	Apr	Aug	Nov
Acinetobacter guillouiae	0.188	0.020	0.005
Acinetobacter johnsonii	0.020	0.071	0.008
Acinetobacter lwoffii_B	0.005	0.012	0.022
Aerococcus urinaeequi	0.024	0.004	0.054
Bifidobacterium pseudolongum_A	0.021	0.002	0.021
CAG-791 sp900101015	0.021	0.023	0.195
CAG-791 sp900318375	0.006	0.005	0.030
CAG-791 sp900320325	0.009	0.003	0.048
Corynebacterium marinum	0.009	0.022	0.036
Corynebacterium xerosis	0.013	0.023	0.228
Cutibacterium acnes	0.020	0.017	0.005
Facklamia_A sp003521095	0.001	0.002	0.035
Jeotgalicoccus sp003513765	0.003	0.005	0.066
Kocuria atrinae	0.001	0.001	0.030
Kocuria salsicia	0.008	0.108	0.123
Kocuria sp002295155	0.002	0.006	0.031
Microbacterium maritypicum	0.000	0.002	0.084
Paracoccus sp000787695	0.002	0.004	0.004
Pseudoclavibacter helvolus	0.011	0.003	0.010
Pseudomonas_E fluorescens_N	0.038	0.000	0.004
Pseudomonas_E lactis	0.038	0.001	0.016
Psychrobacter sp001652315	0.004	0.004	0.038
Psychrobacter sp002352555	0.009	0.000	0.088
Ralstonia insidiosa	0.001	0.000	0.000
Rhodococcus erythropolis_D	0.000	0.001	0.046
Rhodococcus qingshengii	0.002	0.021	0.025
RUG420 sp900317985	0.004	0.002	0.029

TABLE S4. Relative abundances of species that differed by sampling location (locations A to D).

Species	Sampling Location			
	A	B	C	D
Acinetobacter albensis	0.025	0.270	0.016	0.213
Acinetobacter guillouiae	0.020	0.242	0.005	0.001
Acinetobacter lwoffii_B	0.020	0.008	0.009	0.018
Aerococcus urinaeequi	0.062	0.012	0.021	0.008
Anaplasma phagocytophilum	1.414	1.252	1.561	1.208
Bacillus_AM sp001989355	0.042	0.034	0.048	0.032
Brachybacterium nesterenkovii	0.074	0.002	0.038	0.001
CAG-791 sp900101015	0.139	0.032	0.086	0.045
CAG-791 sp900318375	0.024	0.005	0.016	0.005
CAG-791 sp900320325	0.037	0.008	0.021	0.010
Clostridium_F botulinum_A	0.145	0.106	0.147	0.079
Corynebacterium marinum	0.018	0.028	0.025	0.012
Enterococcus faecalis	0.036	0.004	0.008	0.001
Escherichia coli_D	0.021	0.015	0.020	0.009
Kocuria salsicia	0.243	0.018	0.029	0.010
Lactobacillus_C paracasei	0.031	0.001	0.008	0.002
Microbacterium esteraromaticum_A	0.017	0.015	0.021	0.011
Microbacterium sp001425645	0.010	0.058	0.004	0.001
Murimonas intestini	0.544	0.517	0.591	0.585
Mycolicibacterium malmesburyense	0.014	0.011	0.016	0.007
Paracoccus sp000787695	0.008	0.001	0.003	0.001

Pseudomonas_E fluorescens_N	0.004	0.047	0.000	0.002
Psychrobacter sp002352555	0.013	0.073	0.021	0.019
RUG420 sp900317985	0.022	0.004	0.012	0.006

TABLE S5. Climate data retrieved from the Irish Meteorological Service from the sampling days at the four sampling locations. Samples are from farms from 3 different sampling months – April (Apr), August (Aug) and November (Nov) from 4 different sampling locations (A to D).

**Sampling Month**	**Location**	**Rainfall (mm)**	**Max Temp (°C)**	**Min Temp (°C)**	**Grass Min Temp (°C)**	**Mean Wind Speed (knots)**
Apr	A	2.5	13.1	9.9	8.1	11
	B	3.9	12.6	7.3	5	10.6
	C	7.2	14.2	7.5	4.3	6
	D	3.7	13.4	7.6	6.5	7.2
Aug	A	0.1	17.4	10.7	4.8	6.8
	B	0	18.6	10.5	6.4	6.1
	C	0	21.9	8.5	3.9	4.4
	D	0	20.3	7.8	5	3.5
Nov	A	8.2	9.6	3.7	3.3	18.6
	B	15.2	6.0	2.2	1	9.8
	C	18.4	5.7	0.1	-1.6	4.4
	D	15.8	5.2	0.6	-2.5	6.5

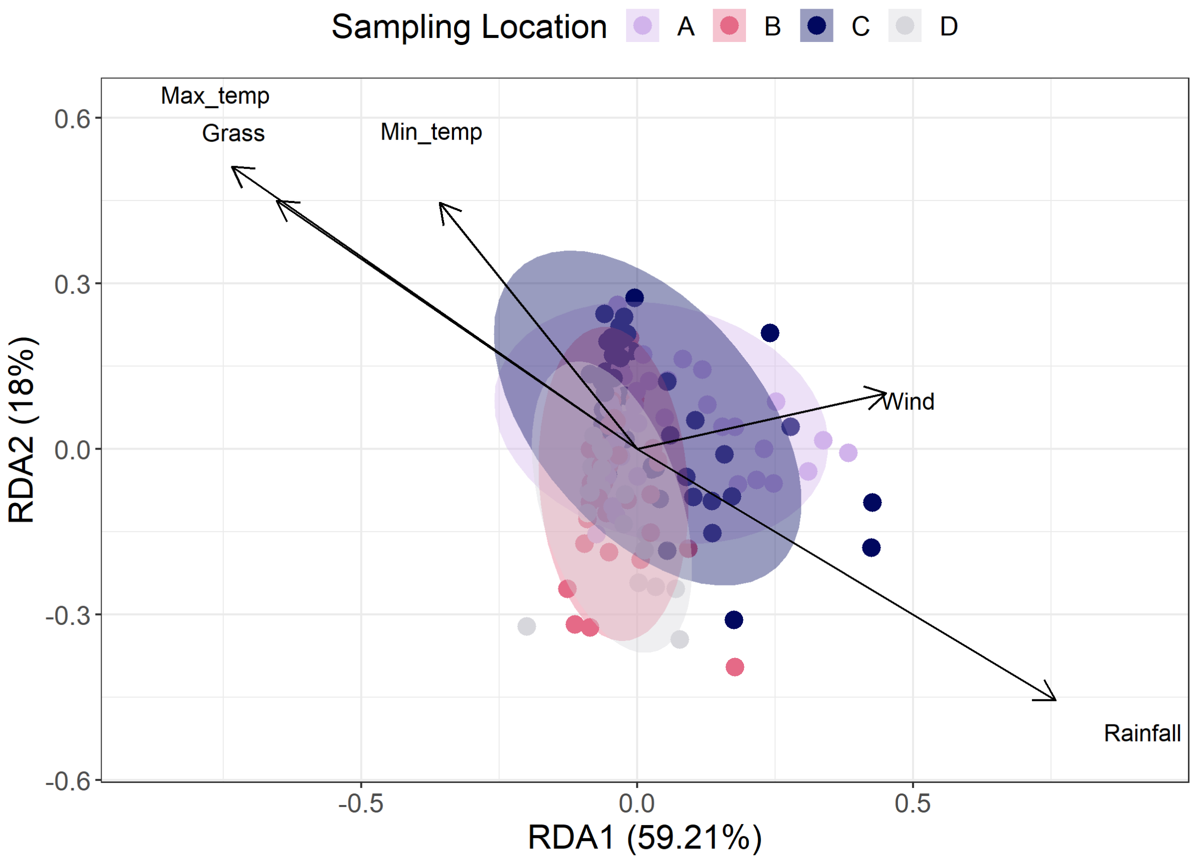

FIG S3. Redundancy analysis of the relationship between climate factors and sampling locations of the bulk tank milk microbiota. The variables are described in Table S5. Ellipses represent clustering by the 4 different sampling locations (A to D).

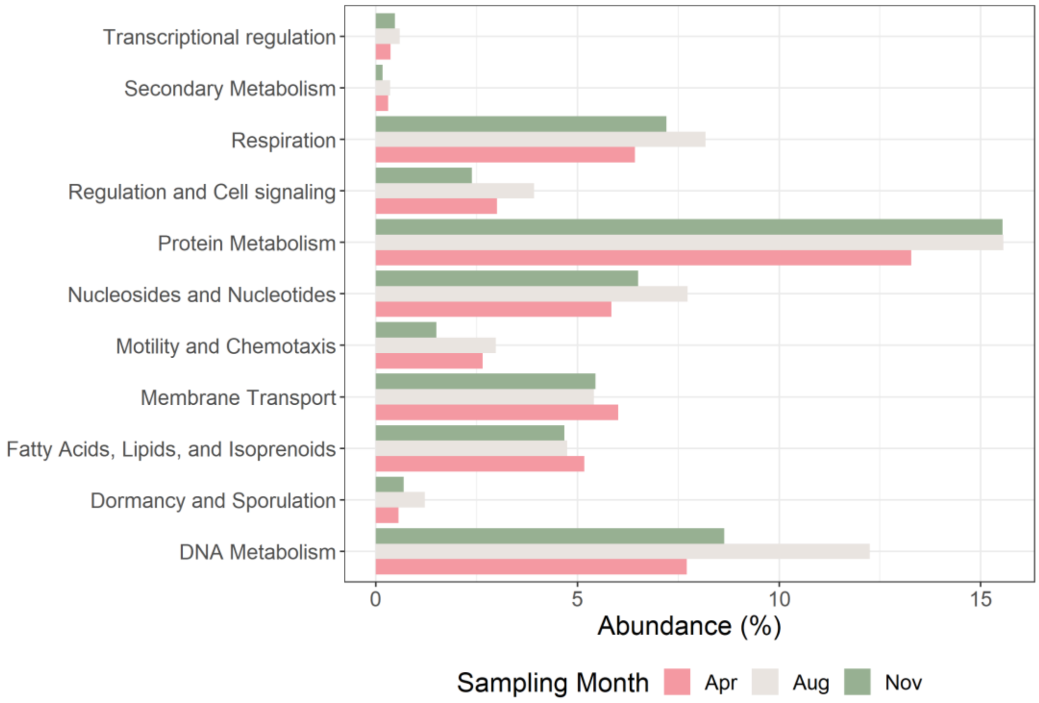

FIG S4. Significantly differential abundances of functions that differed between the sampling months, April (Apr), August (Aug) and November (Nov).

TABLE S6. Relative abundances of genes assigned to significant functional classifications of SUPER-FOCUS subsystem levels 1-3 between sampling months – April (Apr), August (Aug) and November (Nov).

Subsystem Level 1	Subsystem Level 2	Subsystem Level 3	Sampling Month		
			Apr	Aug	Nov
Amino Acids and Derivatives	Alanine, serine, and glycine	-	0.658	0.764	0.814
Amino Acids and Derivatives	Alanine, serine, and glycine	Glycine and Serine Utilization	0.258	0.336	0.338
Amino Acids and Derivatives	Alanine, serine, and glycine	Glycine cleavage system	0.085	0.090	0.130
Amino Acids and Derivatives	Arginine; urea cycle, polyamines	Urea decomposition	0.155	0.214	0.097
Amino Acids and Derivatives	Branched-chain amino acids	-	1.537	1.461	1.242
Amino Acids and Derivatives	Lysine, threonine, methionine, and cysteine	Methionine Degradation	0.335	0.322	0.443
Carbohydrates	-	-	20.429	19.438	19.236
Carbohydrates	-	Putative sugar ABC transporter (ytf cluster)	0.049	0.017	0.019
Carbohydrates	Central carbohydrate metabolism	Dehydrogenase complexes	0.323	0.263	0.232
Carbohydrates	Central carbohydrate metabolism	Glycolysis and Gluconeogenesis	0.417	0.463	0.603

Carbohydrates	Central carbohydrate metabolism	Pyruvate:ferredoxin oxidoreductase	0.110	0.131	0.175
Carbohydrates	CO2 fixation	-	0.473	0.407	0.331
Carbohydrates	CO2 fixation	Photorespiration (oxidative C2 cycle)	0.104	0.086	0.049
Carbohydrates	Di- and oligosaccharides	Fructooligosaccharides(FOS) and Raffinose Utilization	0.237	0.209	0.297
Carbohydrates	Di- and oligosaccharides	Lactose utilization	0.134	0.156	0.190
Carbohydrates	Fermentation	Acetone Butanol Ethanol Synthesis	0.002	0.001	0.003
Carbohydrates	Monosaccharides	Deoxyribose and Deoxynucleoside Catabolism	0.213	0.194	0.276
Carbohydrates	One-carbon Metabolism	millsd methanogensis	0.007	0.009	0.022
Carbohydrates	Organic acids	Glycerate metabolism	0.111	0.083	0.109
Carbohydrates	Polysaccharides	-	0.645	0.794	0.833
Carbohydrates	Sugar alcohols	Glycerol fermentation to 1,3-propanediol	0.011	0.009	0.032
Carbohydrates	Sugar alcohols	Propanediol utilization	0.019	0.011	0.024
Cell Division and Cell Cycle	-	Control of cell elongation - division cycle in Bacilli	0.057	0.031	0.036

Cell Wall and Capsule	Capsular and extracellular polysacchrides	CMP-N-acetylneuraminate Biosynthesis	0.027	0.021	0.037
Cell Wall and Capsule	Capsular and extracellular polysacchrides	Phosphorylcholine incorporation in LPS	0.006	0.007	0.012
Cell Wall and Capsule	Capsular and extracellular polysacchrides	Polysaccharide deacetylases	0.016	0.021	0.035
Cell Wall and Capsule	Gram-Positive cell wall components	D-Alanyl Lipoteichoic Acid Biosynthesis	0.012	0.004	0.004
Cell Wall and Capsule	Gram-Positive cell wall components	Teichuronic acid biosynthesis	0.017	0.018	0.041
Clustering-based subsystems	-	CBSS-262719.3.peg.410	0.075	0.049	0.104
Clustering-based subsystems	-	LMPTP YwlE cluster	0.080	0.080	0.116
Clustering-based subsystems	-	Sporulation-related Hypotheticals	0.014	0.013	0.022
Clustering-based subsystems	contains Thr-tRNA-syn, pyridoxine biosyn, lipid A biosyn, 3 hypos	-	0.007	0.020	0.038
Clustering-based subsystems	contains Thr-tRNA-syn, pyridoxine biosyn, lipid A biosyn, 3 hypos	CBSS-1806.1.peg.1285	0.007	0.020	0.038
Clustering-based subsystems	Protein export?	-	0.253	0.221	0.334

Clustering-based subsystems	Protein export?	CBSS-393121.3.peg.2760	0.253	0.221	0.334
Clustering-based subsystems	Putative Isoquinoline 1-oxidoreductase subunit	-	0.037	0.014	0.017
Clustering-based subsystems	Putative Isoquinoline 1-oxidoreductase subunit	CBSS-314267.3.peg.390	0.037	0.014	0.017
Clustering-based subsystems	Putrescine/GABA utilization cluster-temporal,to add to SSs	-	0.050	0.037	0.019
Clustering-based subsystems	Putrescine/GABA utilization cluster-temporal,to add to SSs	GABA and putrescine metabolism from cluters	0.050	0.037	0.019
Clustering-based subsystems	recX and regulatory cluster	-	0.012	0.003	0.013
Clustering-based subsystems	Sulfatases and sulfatase modifying factor 1 (and a hypothetical)	-	0.110	0.049	0.056
Clustering-based subsystems	Sulfatases and sulfatase modifying factor 1 (and a hypothetical)	Sulfatases and sulfatase modifying factor 1	0.110	0.049	0.056
Cofactors, Vitamins, Prosthetic Groups, Pigments	Coenzyme M	-	0.009	0.003	0.012
Cofactors, Vitamins, Prosthetic Groups, Pigments	Folate and pterines	p-Aminobenzoyl-Glutamate Utilization	0.027	0.013	0.038

Cofactors, Vitamins, Prosthetic Groups, Pigments	Riboflavin, FMN, FAD	Flavodoxin	0.035	0.013	0.034
Cofactors, Vitamins, Prosthetic Groups, Pigments	Tetrapyrroles	Cobalamin	0.107	0.077	0.129
DNA Metabolism	-	-	5.762	6.259	6.506
DNA Metabolism	DNA repair	-	3.087	3.426	3.502
DNA Metabolism	DNA repair	DNA repair, UvrABC system	0.663	0.695	0.853
DNA Metabolism	DNA replication	-	1.561	1.672	1.805
DNA Metabolism	DNA replication	DNA replication, archaeal	0.050	0.046	0.067
DNA Metabolism	DNA replication	DNA topoisomerases, Type II, ATP-dependent	0.292	0.355	0.408
Dormancy and Sporulation	-	-	0.212	0.241	0.279
Dormancy and Sporulation	-	Spore Core Dehydration	0.015	0.008	0.026
Dormancy and Sporulation	-	Sporulation Cluster	0.026	0.029	0.037
Fatty Acids, Lipids, and Isoprenoids	-	-	3.329	3.158	2.930
Fatty Acids, Lipids, and Isoprenoids	Fatty acids	-	1.744	1.536	1.356
Fatty Acids, Lipids, and Isoprenoids	Fatty acids	Fatty Acid Biosynthesis FASII	1.203	1.057	0.936

Fatty Acids, Lipids, and Isoprenoids	Isoprenoids	Isoprenoid Biosynthesis	0.176	0.203	0.230
Fatty Acids, Lipids, and Isoprenoids	Isoprenoids	Isoprenoid Biosynthesis: Interconversions	0.018	0.010	0.030
Iron acquisition and metabolism	-	Heme, hemin uptake and utilization systems in GramPositives	0.113	0.094	0.154
Iron acquisition and metabolism	Siderophores	Petrobactin-mediated iron uptake system	0.018	0.018	0.052
Iron acquisition and metabolism	Siderophores	Siderophore Pyoverdine	0.123	0.057	0.042
Membrane Transport	-	-	3.665	3.410	3.105
Membrane Transport	Sugar Phosphotransferase Systems, PTS	-	0.033	0.032	0.025
Membrane Transport	-	Transport of Manganese	0.106	0.071	0.035
Metabolism of Aromatic Compounds	Metabolism of central aromatic intermediates	N-heterocyclic aromatic compound degradation	0.035	0.014	0.016
Miscellaneous	-	EC 2.7.1.- Phosphotransferases with an alcohol group as acceptor	0.043	0.024	0.030

Miscellaneous	-	EC 6.3.4.- Ligases that form carbon-nitrogen bonds	0.265	0.216	0.275
Miscellaneous	-	Nicotinate catabolism, anaerobic	0.005	0.002	0.012
Miscellaneous	Plant-Prokaryote comparative genomics	COG0277	0.039	0.020	0.021
Miscellaneous	Plant-Prokaryote comparative genomics	Omega-amidase KE2	0.608	0.609	0.743
Motility and Chemotaxis	-	-	0.867	0.762	0.632
Motility and Chemotaxis	Flagellar motility in Prokaryota	-	0.442	0.389	0.303
Motility and Chemotaxis	Flagellar motility in Prokaryota	Flagellum	0.255	0.218	0.170
Nitrogen Metabolism	-	Allantoin Utilization	0.039	0.023	0.032
Nitrogen Metabolism	-	Ammonia assimilation	0.442	0.424	0.344
Nitrogen Metabolism	-	Nitrosative stress	0.041	0.053	0.068
Nucleosides and Nucleotides	-	-	3.820	4.248	4.509
Nucleosides and Nucleotides	-	Pseudouridine Metabolism	0.013	0.012	0.030
Nucleosides and Nucleotides	Purines	-	2.042	2.304	2.469
Nucleosides and Nucleotides	Purines	De Novo Purine Biosynthesis	0.793	0.904	1.055
Nucleosides and Nucleotides	Pyrimidines	-	1.113	1.252	1.281
Nucleosides and Nucleotides	Pyrimidines	De Novo Pyrimidine Synthesis	0.644	0.768	0.824

Phages, Prophages, Transposable elements, Plasmids	Plasmid related functions	-	0.022	0.015	0.024
Phages, Prophages, Transposable elements, Plasmids	Plasmid related functions	Rolling-circle replication	0.021	0.014	0.023
Phosphorus Metabolism	-	Phosphonate metabolism	0.020	0.013	0.014
Protein Metabolism	-	-	9.280	9.651	10.547
Protein Metabolism	Protein biosynthesis	-	6.541	6.849	7.776
Protein Metabolism	Protein biosynthesis	Ribosome LSU bacterial	0.569	0.671	0.754
Protein Metabolism	Protein biosynthesis	Ribosome SSU bacterial	0.349	0.407	0.511
Protein Metabolism	Protein biosynthesis	Single-copy ribosomal proteins	0.579	0.584	0.706
Protein Metabolism	Protein biosynthesis	Translation elongation factor G family	0.155	0.135	0.218
Protein Metabolism	Protein biosynthesis	Translation elongation factors bacterial	0.211	0.213	0.295
Protein Metabolism	Protein biosynthesis	tRNA aminoacylation, Asp and Asn	0.196	0.230	0.261
Protein Metabolism	Protein biosynthesis	Universal GTPases	0.697	0.754	0.900
Protein Metabolism	Protein processing and modification	FeFe hydrogenase maturation	0.017	0.025	0.048

Protein Metabolism	Protein processing and modification	N-linked Glycosylation in Bacteria	0.152	0.166	0.217
Protein Metabolism	Protein processing and modification	SCIFF peptide maturase system	0.010	0.020	0.027
Regulation and Cell signaling	-	-	2.016	1.816	1.628
Regulation and Cell signaling	-	cAMP signaling in bacteria	0.546	0.501	0.355
Regulation and Cell signaling	-	HPr catabolite repression system	0.042	0.039	0.043
Regulation and Cell signaling	-	Sex pheromones in Enterococcus faecalis and other Firmicutes	0.107	0.079	0.132
Respiration	-	-	4.500	4.582	4.195
Respiration	-	Biogenesis of cbb3-type cytochrome c oxidases	0.032	0.020	0.031
Respiration	-	Quinone oxidoreductase family	0.092	0.058	0.032
Respiration	ATP synthases	-	0.506	0.577	0.701
Respiration	ATP synthases	V-Type ATP synthase	0.070	0.104	0.190
Respiration	Electron donating reactions	-	1.785	1.836	1.510
Respiration	Electron donating reactions	CO Dehydrogenase	0.213	0.118	0.074
Respiration	Electron donating reactions	Na(+)-translocating NADH-quinone oxidoreductase and	0.143	0.166	0.266

		rnf-like group of electron transport complexes			
Respiration	Electron donating reactions	Respiratory Complex I	0.731	0.793	0.402
Respiration	Reverse electron transport	-	0.010	0.014	0.015
Respiration	Reverse electron transport	Archaeal membrane bound hydrogenases	0.005	0.007	0.008
Respiration	Reverse electron transport	Energy conserving hydrogenase b, Methanococcales-Methanobacteriales-Methanopyrales	0.002	0.001	0.005
Respiration	Sodium Ion-Coupled Energetics	-	0.034	0.075	0.058
Respiration	Sodium Ion-Coupled Energetics	Na+ translocating decarboxylases and related biotin-dependent enzymes	0.027	0.075	0.057
RNA Metabolism	RNA processing and modification	Polyadenylation bacterial	0.113	0.205	0.193
RNA Metabolism	RNA processing and modification	t(6)A synthesis in bacteria	0.014	0.014	0.025

RNA Metabolism	RNA processing and modification	Threonylcarbamoyladenosine	0.017	0.021	0.026
RNA Metabolism	Transcription	RNA polymerase bacterial	0.485	0.572	0.657
Secondary Metabolism	-	-	0.095	0.056	0.052
Secondary Metabolism	Biologically active compounds in metazoan cell defence and differentiation	-	0.045	0.017	0.020
Secondary Metabolism	Biologically active compounds in metazoan cell defence and differentiation	Steroid sulfates	0.045	0.017	0.020
Stress Response	Acid stress	-	0.013	0.000	0.009
Stress Response	Acid stress	Glutamate transporter involved in acid tolerance in Streptococcus	0.013	0.000	0.009
Stress Response	Oxidative stress	Oxygen stress response / Human gut microbiome	0.021	0.029	0.037
Stress Response	Oxidative stress	Redox-dependent regulation of nucleus processes	0.078	0.093	0.120
Sulfur Metabolism	Organic sulfur assimilation	Alkanesulfonate assimilation	0.236	0.162	0.145
Transcriptional regulation	-	-	0.161	0.133	0.188
Transcriptional regulation	-	CarD	0.161	0.133	0.188

Virulence	-	Mycobacterium virulence operon involved in an unknown function with a Jag Protein and YidC and YidD	0.075	0.045	0.077
Virulence	-	Mycobacterium virulence operon involved in protein synthesis (SSU ribosomal proteins)	0.206	0.204	0.280
Virulence	Resistance to antibiotics and toxic compounds	-	3.059	2.906	2.555
Virulence	Resistance to antibiotics and toxic compounds	Cadmium resistance	0.014	0.018	0.031
Virulence	Resistance to antibiotics and toxic compounds	Mycobacterial MmpL3 membrane protein cluster	0.005	0.008	0.013
Virulence	Resistance to antibiotics and toxic compounds	Resistance to fluoroquinolones	0.290	0.350	0.400
